# A Pilot Study of the Striatal Dopamine Transporter Levels in Kratom-Dependent and Normal Subjects Using 99mTc-TRODAT-1 Single Photon Emission Computed Tomography-Computed Tomography (SPECT-CT)

**DOI:** 10.7759/cureus.43251

**Published:** 2023-08-10

**Authors:** Norasma Amira Binti Zainudin, Nurul Nadiah Zulkifli, Khadijah Abdul Hamid, Hazlin Hashim, Syahir Mansor

**Affiliations:** 1 Biomedical Imaging, Universiti Sains Malaysia, Penang, MYS; 2 Nuclear Medicine Unit, Universiti Sains Malaysia, Penang, MYS

**Keywords:** 99mtc-trodat-1, addiction, kratom, dopamine transporter (dat), spect/ct

## Abstract

Objective: The study aims to elucidate the effects of kratom addiction on dopamine transporter (DAT) using [2-[[2-[[[3-(4-chlorophenyl)-8-methyl-8-azabicyclo[3.2.1]oct-2-yl]methyl](2-mercaptoethyl)amino]ethyl]amino]ethanethiolato(3-)-N2,N20,S2,S20]oxo-[1R-(exo-exo)]-[^99m^Tc] technetium (^99m^Tc-TRODAT-1) brain single photon emission computed tomography-computed tomography (SPECT-CT) in kratom-dependent and healthy subjects.

Materials and methods: We recruited 12 kratom-dependent subjects and 13 healthy men to participate in this study. Addiction, craving, depression, and cognitive scores were assessed. All subjects received a single bolus injection of ^99m^Tc-TRODAT-1 with 914.1 MBq ± 65.5 of activity (mean ± SD). The brain SPECT-CT images were reconstructed using 3D ordered subset expectation maximization (3D-OSEM) along with attenuation correction (AC), scatter correction (SC), and resolution recovery (RR) with an iteration number of four and a subset of 10. The Cohen’s Kappa interrater-reliability between two raters, the standardized uptake value of body weight (SUV_BW_), and the asymmetrical index percentage (AI%) were evaluated.

Results: Kappa statistics showed a fine agreement of abnormal ^99m^Tc-TRODAT-1 uptake in the striatum region for the kratom-dependent group with the κ value of 0.69 (p = 0.0001), and the percentage of agreement for rater 1 and rater 2 was 56% and 64%, respectively. There was a reduction in average SUV in kratom-dependent subjects compared to healthy control subjects in the left caudate and left striatum (0.938 vs. 1.251, p = 0.014, and 1.055 vs. 1.29, p = 0.036, respectively). There was a significant difference in the AI% of the caudate region between the kratom-dependent group and the normal group (33% vs. 14%, p = 0.019).

Conclusion: Our findings signify that kratom addiction, may cause a change in DAT level and the results can be confirmed using ^99m^Tc-TRODAT-1 SPECT-CT.

## Introduction

Kratom (*Mitragyna speciose*), commonly known as ketum in Malaysia, is a tree-like plant that is indigenous to Southeast Asia, such as Thailand, Malaysia, and the Philippines [1, 2]. *Mytragyna speciose* belongs to the Rubiaceae family, which also contains the genus *Coffea *[3]. Kratom has been used in the Southeast Asian region for decades and has recently drawn significant interest in the Western region and a rise in popularity among Malaysians [4, 5]. In Southeast Asia, kratom is traditionally used to manage opioid dependence, improve work performance, and treat common health issues like hypertension, cough, and fever [2, 5]. Kratom, similar to coffee, contains a variety of alkaloids, some of which are active in the central nervous system (CNS) and can have a variety of physiological and behavioral effects [3]. Mitragynine is the major alkaloid in kratom leaves and is an μ-opioid receptor agonist, which is responsible for analgesic effects [6, 7]. In addition, kratom is also presumed to give effects similar to those of drugs such as stimulants, sedatives, and euphoric feelings to the abuser [5, 8].

Any opiate substance, including heroin, oxycodone, and others, attaches to μ-opioid receptors on the surfaces of opiate-sensitive brain cells as it circulates through the bloodstream to the brain [9]. In medicine, opioids are prescribed to relieve pain; however, when opioids are consumed in the absence of significant pain, they activate the rewarding process in the brain, which triggers the feeling of consuming more opioids for pleasure [10, 11]. Opioids may affect several brain circuits, including the mesolimbic (midbrain) reward system, which results in the release of chemical dopamine (DA) in the nucleus accumbens (NAc), causing a feeling of pleasure [12]. One of the main reasons why some people use drugs repeatedly is because opioids stimulate the brain's reward system, especially in the early stages of abuse. Nevertheless, the need for opioids becomes compelling over time and transcends a purely recreational motivation. Dependence and tolerance both contribute to this heightened compulsion [9].

Dopamine (DA) regulates a wide range of physiological functions, including movement, cognition, reward, and addiction [13]. Dopamine is the neurotransmitter that is associated with the reinforcing effects of addictive substances and plays a significant role in initiating the neurobiological changes related to addiction [14, 15]. The reuptake of DA from synaptic clefts into the presynaptic terminal is carried out by a trans-membrane protein known as dopamine transporter (DAT) [16]. Dopamine transporter has been proposed as the molecular site of action for repressing drug addiction-related mechanisms [17]. Therefore, in several studies regarding drug abuse or addiction, they evaluate the distribution of DAT activity using the uptake radiolabeled substrate such as [2-[[2-[[[3-(4-chlorophenyl)-8-methyl-8-azabicyclo[3.2.1]oct-2-yl]methyl](2-mercaptoethyl)amino]ethyl]amino]ethanethiolato(3-)-N2,N20,S2,S20]oxo-[1R-(exo-exo)]-[99mTc] technetium (^99m^Tc-TRODAT-1) [16, 17]. In drug addictions such as cocaine, heroin, and methamphetamine, there are significant reductions in D_2_ DA receptor availability in the striatum (14). Nevertheless, there is no study for kratom addiction related to the DA receptor availability in the striatum. Therefore, we would like to assess the DAT activity in kratom-dependent subjects, as, in fact, kratom consists of a μ-opioid receptor agonist. The purpose of the study is to compare the effects of kratom addiction on DAT density using ^99m^Tc-TRODAT-1 SPECT-CT brain images in kratom-dependent and healthy subjects.

## Materials and methods

Participants

Twelve kratom-dependent subjects (male, aged 31.58 ± 11.28 years old) volunteered to participate in this study. On the other hand, 13 healthy control subjects, men aged 32.23 ± 6.96 years old, volunteered to participate in this study. Twelve subjects had never smoked, and one was an active smoker. The recorded kratom intake quantity was 2.13 ± 1.68 L/day (range: 0.5-6 L/day), and the mean duration of usage was 9.92 ± 6.54 years (range: 2-21 years). All subjects were active smokers. All subjects underwent urine drug tests to rule out any possibility of illicit drugs being used. All kratom-dependent and healthy subjects had no neurological or psychiatric diseases.

Statement of ethics

The study was approved by the Human Research Ethics Committee (HREC) of Universiti Sains Malaysia (USM), Penang, Malaysia, and a written consent form was obtained from participants who participated in this study (approval number: USM/JEPeM/20020113).

Assessment of kratom addiction, anxiety, and depression

Kratom addiction was assessed using the Diagnostic and Statistical Manual of Mental Disorders, Fifth Edition (DSM-V), which is specifically used for substance disorders. For the subject who had a history of a failed trial of quitting kratom, further withdrawal symptoms were measured using the Hamilton Anxiety Rating Scale (HAM-A). The cognitive function was assessed using the Mini-Mental State Examination (MMSE) test, and the depression was assessed using the Patient Health Questionnaire-9 (PHQ-9) questionnaire.

Magnetic resonance imaging (MRI) scans

All subjects underwent a magnetic resonance imaging (MRI) procedure using a General Electric (GE) Signa HDx 1.5T (GE Healthcare, Wisconsin, USA) device before the SPECT-CT scans. The purpose of this scan was to specify the anatomical structure of the caudate and putamen (striatum) regions [18]. The gradient echo inversion recovery-isotropic 3D T1 imaging (BRAVO) sequence was used with an image voxel size of 512 × 512 × 64. 

Single photon emission computed tomography-computed tomography (SPECT-CT) scans

After the completion of the MRI brain scans, the subjects received a single bolus injection of ^99m^Tc-TRODAT-1 with 914.1 MBq ± 65.5 of activity for all subjects. The brain SPECT-CT images were acquired using the GE Discovery 670 SPECT-CT system (GE Healthcare, Wisconsin, USA) after three to four hours of injection using the step-and-shoot method, 30 s per view, 120 views with a 128 × 128 × 128 image voxel. The SPECT-CT images were reconstructed using 3D-ordered subset expectation maximization (3D-OSEM) along with attenuation correction (AC), scatter correction (SC), and resolution recovery correction (RR) with an iteration number of four and a subset of 10 (4i10s) with a Butterworth filter with a frequency of 0.4 and order of 10 for qualitative evaluation. In addition, for quantitative evaluation, the SPECT-CT was reconstructed using 3D-OSEM along with attenuation, scatter, and resolution recovery correction with an iteration number of four and a subset of 10 (4i10s) without any postfilter. All reconstructed SPECT-CT images were then fused to MRI images using rigid body registration in A Medical Image Data Examiner (AMIDE) version 1.0.4 (GNU General Public Library (GPL), Boston, MA, USA) for image analysis.

Image analysis

Kappa’s Statistic

For qualitative analysis, the post-reconstructed brain SPECT/MRI-fused images were evaluated by two nuclear medicine specialists from the Advanced Medical and Dental Institute at USM. Both specialists were given a form with the details of each subject concealed. They were asked to score each caudate, putamen, and striatum as normal or abnormal. The data scores were analyzed in Minitab Data Analysis Tools software (Minitab, LLC, State College, Pennsylvania, USA) using Kappa statistics to measure the percent of agreement between two raters. The Kappa κ values range from −1 to +1. The higher the value of κ, the stronger the agreement [19]. We evaluated the Kappa value as follows: for interpretation, κ < 0.20 represented a poor agreement, 0.21-0.40 represented a fair agreement, 0.41-0.60 represented a moderate agreement, 0.61-0.80 represented a good agreement, and > 0.81 represented excellent agreement [20]. Furthermore, the percentage of agreement (PA) value was also generated from this test.

Quantitative Analysis

The reconstructed SPECT-CT images were analyzed using AMIDE [21]. All reconstructed SPECT-CT datasets were co-registered onto MRI images to guide the brain's anatomical structure. Several volumes of interest (VOIs) were manually drawn (delineated) in the areas of the caudate, putamen, and occipital region. The uptake from areas of interest (ROI) delineated bilaterally in the striatum, caudate nucleus, putamen, and occipital lobe was used to quantify DAT density [22]. The count concentration was reported as the standardized uptake value of body weight (SUV_BW_).

Then, we also calculate the asymmetrical index (AI) by using the formula: AI% = (R - L)/(R + L) * 100, where R is the striatum on the side that is less severe, and L is the ROI of the striatum that is more severe [23].

## Results

Based on demographic data summarized in Table 1, there were 12 kratom-dependent subjects and 13 healthy control subjects, with the average age for each group being 31.58 ± 11.28 years and 32.23 ± 6.96 years, respectively.

**Table 1:** Demographic data and substance use features of participants with kratom-dependence and healthy controls

	Kratom-dependent mean (SD)	Healthy control mean (SD)	p-value
Gender (male)	12	13	
Age, mean ± SD, years	31.58 (11.28)	32.23 (6.96)	0.862
Body mass index (kg/m2), mean ± SD	24.56 (7.71)	26.81 (5.70)	
Kratom abuse variable			
Age of first use (years)	21.83 (7.65)	-	
Duration of kratom used (years)	9.92 (6.54)		
Quantity of use (L/day)	2.13 (1.68)	-	
Smoking variables, n (%)	12	1	
Activity injected (MBq)	905.11 (74.13)	922.32 (55.12)	0.532

The mean duration of kratom use was 9.92 ± 6.54 years (two to 21 years), and the average age of first use was 21.83 ± 7.65 years old (12-34 years old). The average kratom used per day was 2.13 ± 1.68 L (0.5-6.0 L). There was no significant difference in age (p = 0.862) or injected activity (p = 0.532) between groups.

Neuropsychological performance and clinical ratings in kratom-dependent and healthy control subjects

**Figure 1:**
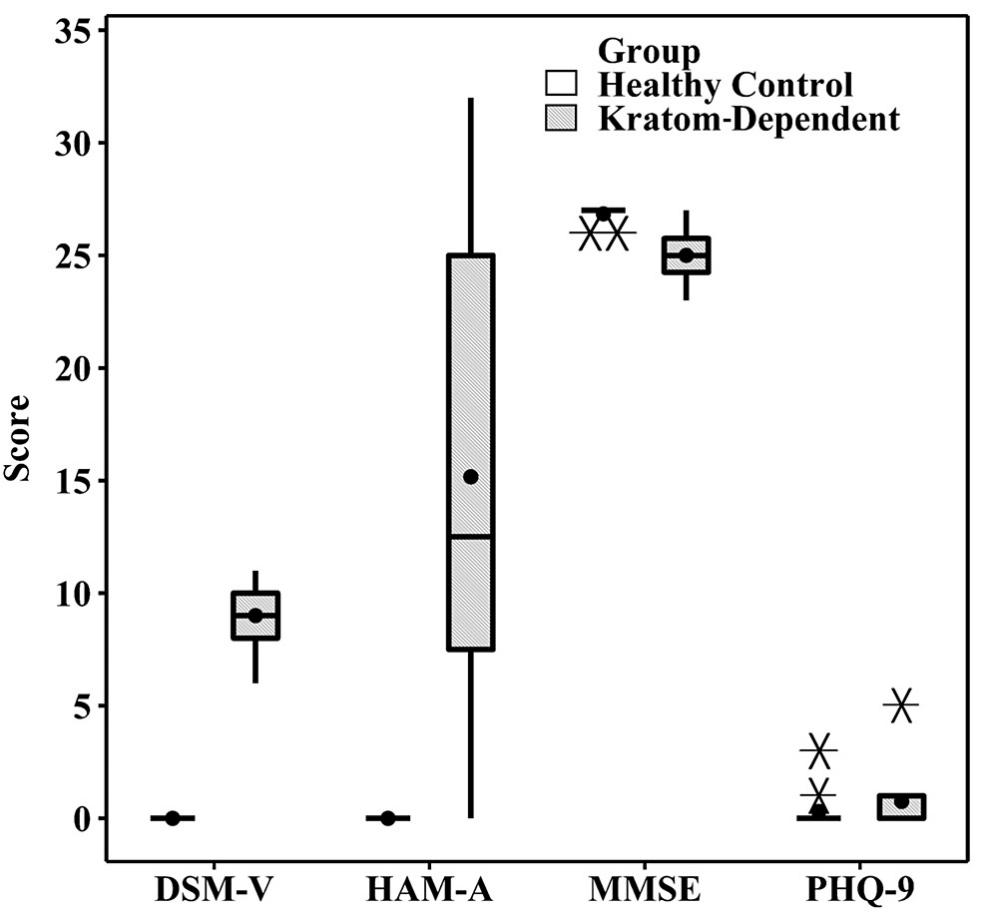
The DSM-V, HAM-A, MMSE, and PHQ scores for kratom-dependent and healthy control subjects DSM-V: The Diagnostic and Statistical Manual of Mental Disorders, Fifth Edition; HAM-A: Hamilton Anxiety Rating Scale; MMSE: Mini-Mental State Examination; PHQ: Patient Health Questionnaire-9. The star (*) symbols represent the outlier data points within the group

Based on Figure 1, the DSM-V score of kratom-dependent subjects shows severe addiction to kratom, with a total mean score of nine.

In addition, the HAM-A anxiety score showed that the majority of kratom-dependent subjects showed mild withdrawal symptoms with a mean score of 15, and only a few of the subjects showed severe withdrawal symptoms (HAM-A score of more than 30). The MMSE score showed normal cognitive function for kratom-dependent subjects (score ≥ 25), and only two subjects showed mild dementia with a mean score of 23. Based on the PHQ-9 screening questionnaire, the result showed none to minimal depression in kratom-dependent subjects. For healthy control subjects, the neuropsychological performance showed a normal score (DSM-V score = 0, HAM-A =0, MMSE < 25, and PHQ-9 score ≤ 3).

Kappa’s statistic

Qualitative assessment by two nuclear medicine physicians had a good agreement that there was abnormal uptake of ^99m^Tc-TRODAT-1 for the kratom-dependent group in the right caudate, right putamen, and left caudate with κ = 0.78 (p = 0.000, PA = 92%), κ = 0.61 (p = 0.0004, PA = 80%), and κ = 0.70 (p = 0.0004, PA = 92%), respectively. The greater the percentage agreement, the lower the resulting value of the kappa (20). Table 2 represents Kappa’s statistical results. 

**Table 2:** Kappa’s statistical result between the two raters SE: standard error

	Κ value	SE Kappa	Z	p (vs > 0)	Percentage Agreement (%)
Right caudate	0.78	0.20	3.90	0	92
Right putamen	0.61	0.18	3.32	0.0004	80
Left caudate	0.70	0.20	3.51	0.0002	92
Left putamen	0.45	0.17	2.70	0.0035	72
Overall	0.69	0.12	3.62	0.0001	84

Based on our finding, for the overall cross-assessment of the SPECT-CT images, both raters finely agreed that the SPECT images showed an abnormal ^99m^Tc-TRODAT-1 uptake in the striatum region for the kratom-dependent group, with a κvalue of 0.69 (p = 0.0001) and a percentage of agreement for raters one and two of 56% and 64%, respectively.

Quantitative analysis

**Figure 2:**
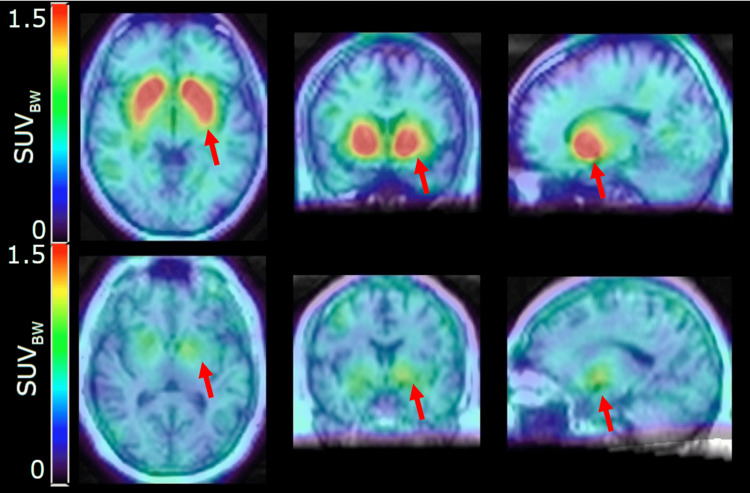
The SUVBW images overlay on the MRI of one of the normal subjects (top row) versus one of the kratom-dependent subjects (bottom row) for axial, coronal, and sagittal slices. Kratom-dependent subjects consumed the kratom for 21 years of duration. Images were reconstructed using 4i10s (Butterworth filter frequency of 0.4 and an order of 10) for image display. Red arrows represent the striatum region for normal subjects and kratom-dependent subjects, respectively. SUV_BW_: standardized uptake value of body weight

Figure 2 represents the SUV_BW_ value of one of the normal subjects compared to a kratom-dependent subject.

There is a distinctive reduction in ^99m^Tc-TRODAT-1 uptake in the striatum region for the kratom-dependent subject. The quantitative value of SUV_BW_, represented in Table 3, shows the SUV in left caudate is significantly low in kratom-dependent subjects with a mean SUV of 0.94 compared to healthy controls with a mean SUV of 1.25 (p = 0.014).

**Table 3:** The SUVBW and AI (%) of kratom-dependent and healthy controls SUV_BW_: standardized uptake value of body weight; AI: asymmetrical index; SD: standard deviation

	Kratom-dependent mean (SD)	Healthy control mean (SD)	p-value
SUV_BW_			
Right caudate	1.25 (0.36)	1.21 (0.3)	0.698
Right putamen	1.16 (0.25)	1.37 (0.31)	0.093
Right striatum	1.2 (0.28)	1.29 (0.29)	0.507
Left caudate	0.94 (0.26)	1.25 (0.31)	0.014
Left putamen	1.17 (0.3)	1.33 (0.28)	0.215
Left striatum	1.06 (0.22)	1.29 (0.28)	0.036
Striatum	1.13 (0.24)	1.29 (0.28)	0.161
Occipital	0.67 (0.13)	0.74 (0.17)	0.242
AI (%)			
Caudate	33 (25.3)	14.4 (11)	0.019
Putamen	10.6 (5.1)	8.5 (3.5)	0.272
Striatum	13.4 (9.2)	8 (4.5)	0.068

**Figure 3:**
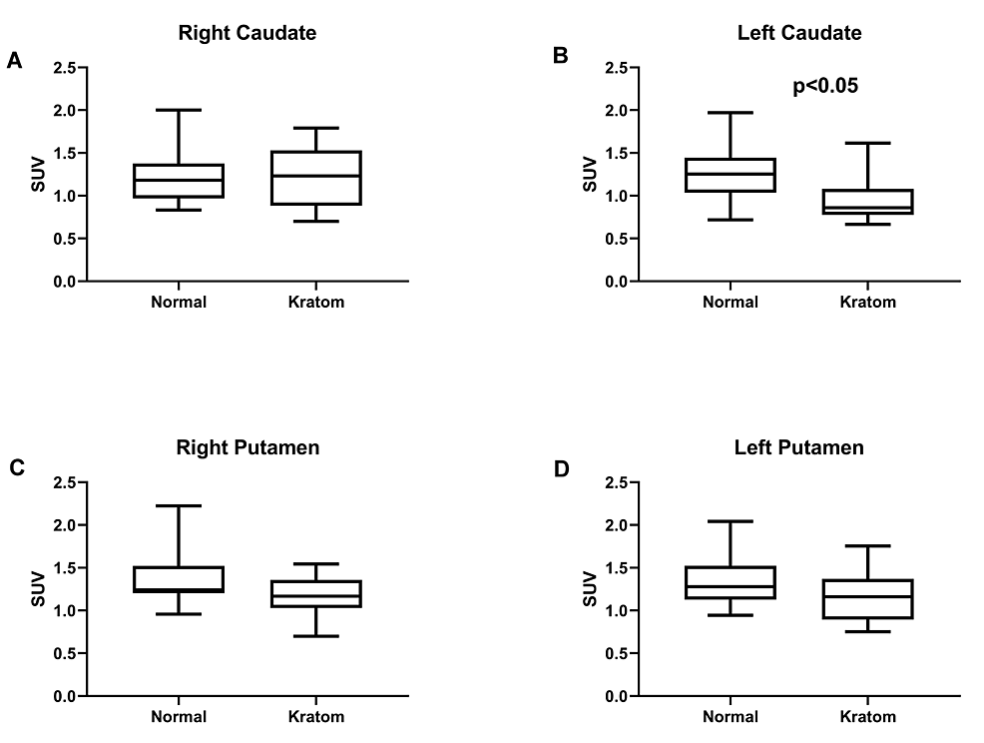
The SUVBW for kratom-dependent and healthy control subjects for right caudate (A), left caudate (B), right putamen (C), and left putamen (D) SUV_BW_: standardized uptake value of body weight

In addition, the SUV for the left striatum was also significantly low in kratom-dependent subjects, with a mean SUV of 1.06 compared to the healthy control subject (mean SUV: 1.29, p = 0.036). Besides that, the right caudate, right putamen, and left putamen also showed a reduction of SUV in the kratom-dependent subject compared to the healthy control subject (SUV_right caudate_ = 1.25 vs. 1.21, SUV_right putamen_ = 1.16 vs. 1.37, and SUV_left putamen_ = 1.17 vs. 1.33), but not significantly, as the p-values were 0.698, 0.093, and 0.215, respectively. Figure 3 depicts a significant reduction of SUV in kratom-dependent subjects compared to healthy control subjects in the left caudate region.

**Figure 4:**
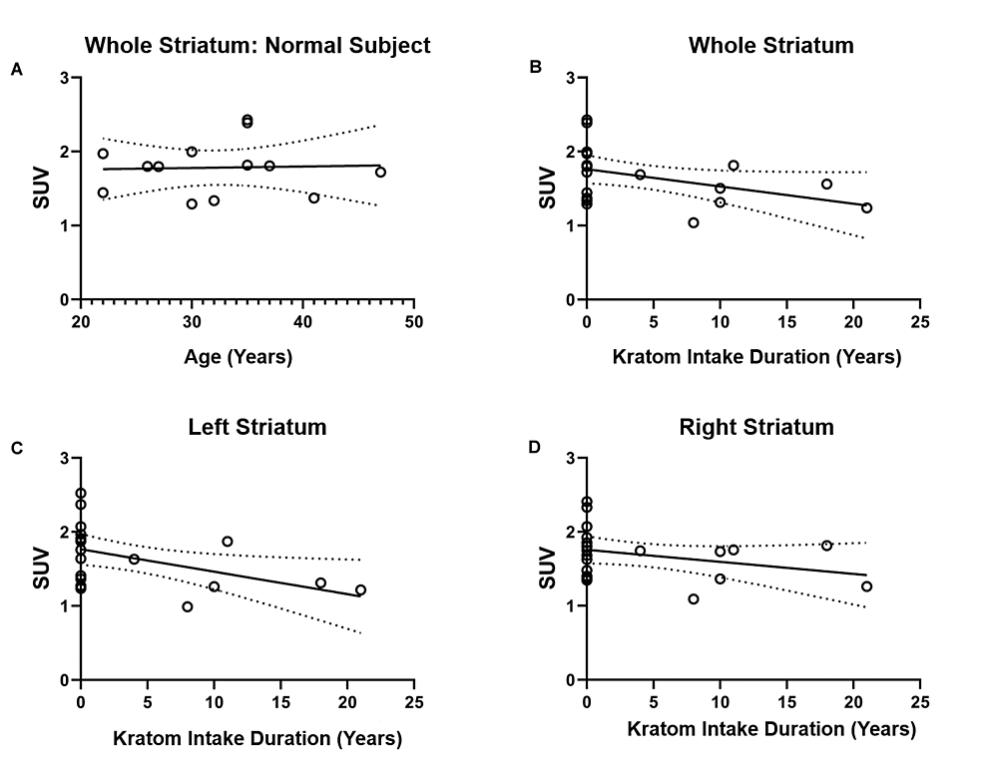
The SUV trend for a healthy control subject for the whole striatum (A), the SUV trend for a kratom-dependent subject for the whole striatum (B), the left striatum (C), and the right striatum (D); the dotted lines show 95% of the confidence interval.

Figure 4 (A) shows no significant SUV trend for the whole striatum in healthy control subjects over the subjects' age.

Furthermore, as shown in Figures 4B-4D, the mean SUV in kratom-dependent subjects decreased with the longer duration of kratom intake.

**Figure 5:**
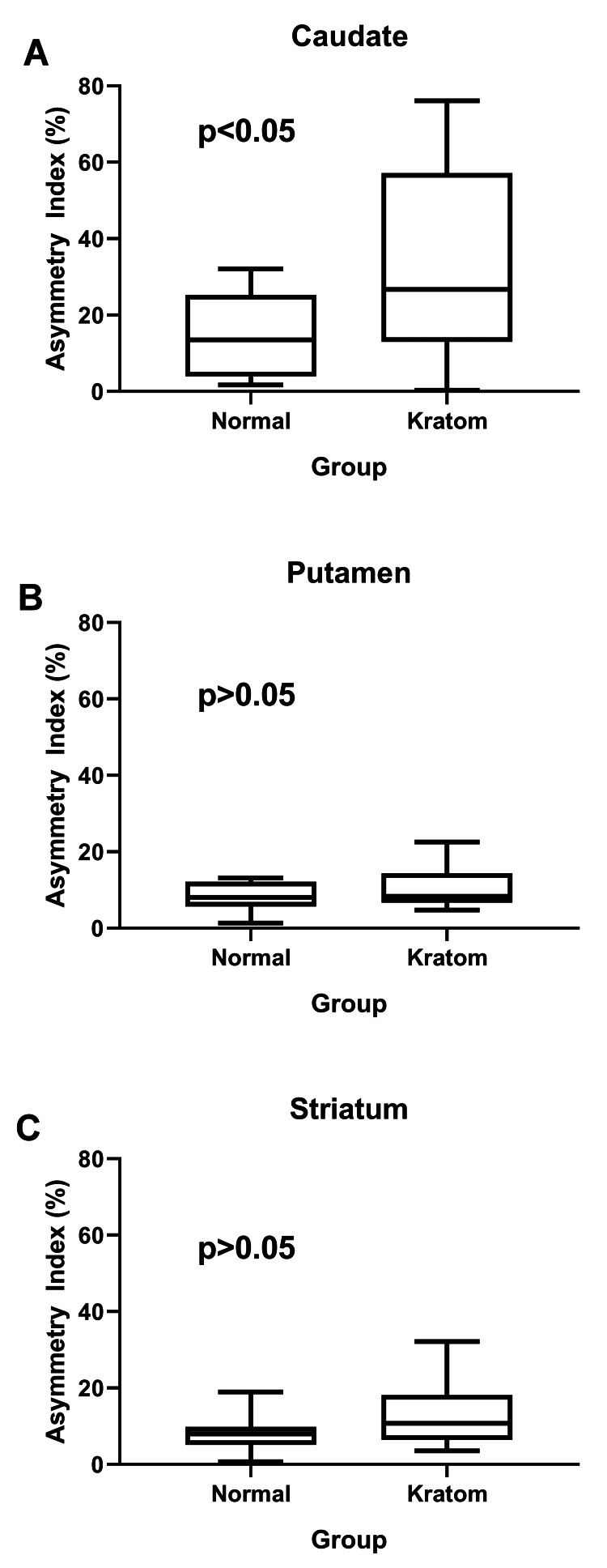
The asymmetrical index percentage (%) for kratom-dependent and healthy control subjects for caudate (A), putamen (B), and striatum (C)

Besides that, the asymmetrical index (AI) in the caudate region was significantly higher in the kratom-dependent subject (AI% = 33.0) compared to the healthy control subject (AI% = 14.4 (p = 0.019), as shown in Table 3 and Figure 5.

**Figure 6:**
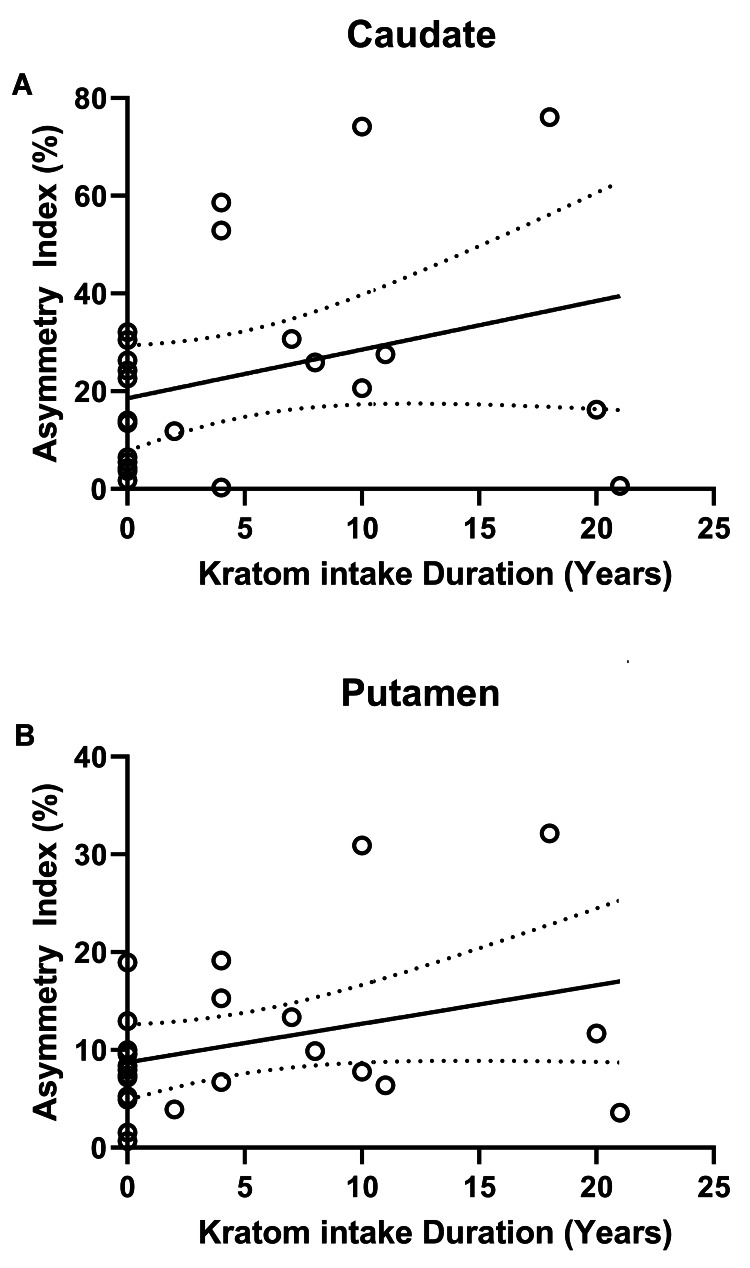
The asymmetrical index trendline for kratom-dependent and healthy control subjects for caudate (A) and putamen (B); the dotted lines show 95% of the confidence interval.

In addition, there was also a refined AI% for the caudate and striatum regions for kratom-dependent subjects (AI% = 10.6 and 13.4, respectively) in contrast to healthy control subjects (AI% = 8.5 and 8.0, respectively). However, it was not significant as the p-values were 0.272 and 0.068 for the kratom-dependent subject and the control healthy subject, respectively. Additionally, as illustrated in Figure 6, the AI% varied proportionally with the kratom intake duration.

## Discussion

Table 1 shows no significant difference in age among groups. Even though based on cognitive theory, the DA diminished with age as the elderly may have a decline in cognitive activities, such as measures of processing speed, working memory, and executive cognitive function, that require speedy information processing or transformation to make a decision [24, 25]. However, in this study, all healthy subjects did not have any abnormal cognitive functions.

The kratom-dependent subjects showed severe addiction and mild withdrawal symptoms (e.g., runny nose, back pain, and headache) depending on the duration of kratom use over the years. The opioid activates the mesolimbic (midbrain) reward system, which generates a signal in the ventral tegmental area (VTA), resulting in the release of the DA in another part of the brain called the nucleus accumbens (NAc), which causes feelings of pleasure [9]. Because of this, they will consume opioids repeatedly; nevertheless, over time, the need for opioids will exceed the pleasure, and they will become dependent (9). The withdrawal effect includes major motivating factors such as persistent irritation, emotional distress, malaise, dysphoria, alexithymia, stressful circumstances, and a lack of enthusiasm for natural rewards, which may be associated with the changes in DAT [10]. Furthermore, the MMSE score for cognitive function for two kratom-dependent subjects shows mild dementia. However, the result might be a false positive and might be referred for further assessment as the MMSE is a paper-based test. Even though the MMSE is part of determining whether or not someone has dementia, the test results should be evaluated in consideration of the patient's personality, behavior, and level of functioning in daily life and at home [26].

Cohen’s Kappa κ value showed good agreement between the two raters, as seen in Table 2. Although the kappa was established to mitigate the possibility of guessing and the assumption by the rater is independence, it may result in an overly lower estimation of agreement [20].

As shown in Table 3 and Figure 3, mean SUV was reduced in kratom-dependent subjects, especially in the left caudate and left striatum regions. A study reported that depressed patients with affective flattening and psychomotor retardation had decreased presynaptic dopamine function in the left caudate. Psychomotor retardation is one of the components in the diagnosis of depressive disorder [27]. In this study, the kratom-dependent subject showed mild to minimal depression. Anyhow, we only did the assessment based on PHQ-9, and we did not evaluate their psychomotor retardation. Furthermore, a lower SUV indicates a lower binding level of ^99m^Tc-TRODAT-1, reflecting greater dopaminergic nerve terminal degeneration and hence lower dopaminergic activity [28]. We know that kratom consists of mitragynine, and uncontrolled use in the long term may impair the brain’s dopaminergic system. Likewise, there are a few other substances of abuse, such as methamphetamine, cocaine, and alcohol, which can have distinct effects on how DAT is regulated [29]. For example, methamphetamine is associated with decreased DAT density, as shown by positron emission tomography (PET) imaging and DAT ligands [30, 31]. Besides that, long-term kratom intake may also decrease the DAT level. Other drug abuse studies reported that the long-term effects of cocaine abuse caused the dopamine transporters or receptors to become more or less abundant on nerve cell surfaces [32]. In addition, cocaine's long-term behavioral impacts could be correlated to changes in dopamine transmission that result from DAT blockage [33]. Chronic cocaine usage is linked to a reduction in dopamine D2 receptor availability due to high levels of synaptic dopamine, which lasts three-four months after detoxification [14, 33].

The kratom-dependent group had a higher AI% in the caudate region compared to the healthy control group. The AI% can be used as an indicator to evaluate the striatal asymmetry-reduced^ 123^I-Iofupane accumulation on DAT-SPECT [23]. Based on the previous study, the AI% values were significantly higher in Parkinson’s disease (PD) than in the control group [23, 34]. From the results of our study, we could say that as the value of SUVs decreased, the value of AI% increased. The AI% alone is not a concrete indicator, as one of the kratom-dependent subjects shows a significant reduction in caudate and putamen uptake with a low AI%. The low AI% is due to the identical value of uptake between both left and right, for caudate and putamen.

However, there are several limitations to our study. Firstly, the sample size was small and limited in regard to the age group. Furthermore, to our knowledge, this is the first study to assess the DAT level using ^99m^Tc-TRODAT-1 in kratom-abused subjects. The study may be expanded to a large population with numerous age groups in the future. Future studies should examine the confounding influence of nicotine exposure on kratom dependence, as it is impossible to have kratom-abused subjects who are not smoking.

## Conclusions

Our findings from the pilot study signify that kratom addiction may cause a change in DAT level with similar effects to other opiate drugs such as heroin, morphine, and codeine. The duration of kratom consumption may disrupt striatal dopamine transporter neuronal activity, but more data are required to increase the statistical power of the data. A SPECT-CT with ^99m^Tc-TRODAT-1 radiopharmaceutical could be a useful tool in stratifying the level of DAT in kratom-dependent subjects.

## References

[1] Prozialeck WC, Avery BA, Boyer EW (2019). Kratom policy: the challenge of balancing therapeutic potential with public safety. Int J Drug Policy.

[2] Eastlack SC, Cornett EM, Kaye AD (2020). Kratom-pharmacology, clinical implications, and outlook: a comprehensive review. Pain Ther.

[3] Patay ÉB, Bencsik T, Papp N (2016). Phytochemical overview and medicinal importance of Coffea species from the past until now. Asian Pac J Trop Med.

[4] Henningfield JE, Fant RV, Wang DW (2018). The abuse potential of kratom according the 8 factors of the controlled substances act: implications for regulation and research. Psychopharmacology (Berl).

[5] Mallow MS (2020). Ketum abuse in Malaysia: its legal status and proposed solution. Perdana: International Journal of Academic Research.

[6] Kamble SH, León F, King TI (2020). Metabolism of a kratom alkaloid metabolite in human plasma increases its opioid potency and efficacy. ACS Pharmacol Transl Sci.

[7] Todd DA, Kellogg JJ, Wallace ED (2020). Chemical composition and biological effects of kratom (Mitragyna speciosa): In vitro studies with implications for efficacy and drug interactions. Sci Rep.

[8] Gibbons S, Arunotayanun W (2013). Chapter 14 - natural product (fungal and herbal) novel psychoactive substances. Novel Psychoactive Substances: Classification, Pharmacology and Toxicology.

[9] Kosten TR, George TP (2002). The neurobiology of opioid dependence: implications for treatment. Sci Pract Perspect.

[10] Koob GF, Volkow ND (2016). Neurobiology of addiction: a neurocircuitry analysis. Lancet Psychiatry.

[11] Veltri C, Grundmann O (2019). Current perspectives on the impact of kratom use. Subst Abuse Rehabil.

[12] Tomkins DM, Sellers EM (2001). Addiction and the brain: the role of neurotransmitters in the cause and treatment of drug dependence. CMAJ.

[13] Zhu J, Reith ME (2008). Role of the dopamine transporter in the action of psychostimulants, nicotine, and other drugs of abuse. CNS Neurol Disord Drug Targets.

[14] Volkow ND, Fowler JS, Wang GJ, Swanson JM, Telang F (2007). Dopamine in drug abuse and addiction: results of imaging studies and treatment implications. Arch Neurol.

[15] Lüscher C, Ungless MA (2006). The mechanistic classification of addictive drugs. PLoS Med.

[16] Yen CH, Yeh YW, Liang CS (2015). Reduced dopamine transporter availability and neurocognitive deficits in male patients with alcohol dependence. PLoS One.

[17] Ciliax BJ, Heilman C, Demchyshyn LL (1995). The dopamine transporter: immunochemical characterization and localization in brain. J Neurosci.

[18] Dickson J, Ross J, Vöö S (2019). Quantitative SPECT: the time is now. EJNMMI Phys.

[19] (2022). Example of attribute agreement analysis. https://support.minitab.com/en-us/minitab/21/help-and-how-to/quality-and-process-improvement/measurement-system-analysis/how-to/attribute-agreement-analysis/attribute-agreement-analysis/before-you-start/example/.

[20] McHugh ML (2012). Interrater reliability: the kappa statistic. Biochem Med (Zagreb).

[21] (2022). AMIDE: Amide's a Medical Imaging Data Examiner. http://amide.sourceforge.net/..

[22] Bor-Seng-Shu E, Felicio AC, Braga-Neto P (2014). Dopamine transporter imaging using 99mTc-TRODAT-1 SPECT in Parkinson's disease. Med Sci Monit.

[23] Shigekiyo T, Arawaka S (2020). Laterality of specific binding ratios on DAT-SPECT for differential diagnosis of degenerative parkinsonian syndromes. Sci Rep.

[24] Murman DL (2015). The impact of age on cognition. Semin Hear.

[25] Karrer TM, Josef AK, Mata R, Morris ED, Samanez-Larkin GR (2017). Reduced dopamine receptors and transporters but not synthesis capacity in normal aging adults: a meta-analysis. Neurobiol Aging.

[26] Creavin ST, Wisniewski S, Noel-Storr AH (2016). Mini-Mental State Examination (MMSE) for the detection of dementia in clinically unevaluated people aged 65 and over in community and primary care populations. Cochrane Database Syst Rev.

[27] Bains N, Abdijadid S (2023). Major Depressive Disorder.

[28] Voon V, Rizos A, Chakravartty R (2014). Impulse control disorders in Parkinson's disease: decreased striatal dopamine transporter levels. J Neurol Neurosurg Psychiatry.

[29] Yuan J, Liu XD, Han M, Lv RB, Wang YK, Zhang GM, Li Y (2017). Comparison of striatal dopamine transporter levels in chronic heroin-dependent and methamphetamine-dependent subjects. Addict Biol.

[30] McCann UD, Wong DF, Yokoi F, Villemagne V, Dannals RF, Ricaurte GA (1998). Reduced striatal dopamine transporter density in abstinent methamphetamine and methcathinone users: evidence from positron emission tomography studies with [11C]WIN-35,428. J Neurosci.

[31] Volkow ND, Chang L, Wang GJ (2001). Loss of dopamine transporters in methamphetamine abusers recovers with protracted abstinence. J Neurosci.

[32] Nestler EJ (2005). The neurobiology of cocaine addiction. Sci Pract Perspect.

[33] Crits-Christoph P, Newberg A, Wintering N (2008). Dopamine transporter levels in cocaine dependent subjects. Drug Alcohol Depend.

[34] Lin W, Zuo CT, Wu JJ, Yang LK, Zhu J, Wang YH (2022). Striatal asymmetry index and its correlation with the Hoehn & Yahr stage in Parkinson's disease. Int J Neurosci.

